# Ten simple rules for pushing boundaries of inclusion at academic events

**DOI:** 10.1371/journal.pcbi.1011797

**Published:** 2024-03-01

**Authors:** Siobhan Mackenzie Hall, Daniel Kochin, Carmel Carne, Patricia Herterich, Kristen Lenay Lewers, Mohamed Abdelhack, Arun Ramasubramanian, Juno Felecia Michael Alphonse, Visotheary Ung, Sara El-Gebali, Christopher Brian Currin, Esther Plomp, Rachel Thompson, Malvika Sharan

**Affiliations:** 1 Nuffield Department of Surgical Sciences, Medical Sciences Division, University of Oxford, Oxford, United Kingdom; 2 Deep Learning Indaba, London, United Kingdom; 3 Centre for Human Genetics, University of Oxford, Oxford, United Kingdom; 4 Independent Researcher, Cape Town, South Africa; 5 Information Science, University of Colorado Boulder, Boulder, Colorado, United States of America; 6 Krembil Centre for Neuroinformatics, Centre for Addiction and Mental Health, Toronto, Ontario, Canada; 7 Arabs in Neuroscience, Toronto, Ontario, Canada; 8 Department of Education, University of Oxford, Oxford, United Kingdom; 9 ISYEB UMR 7205 Centre National de la Recherche Scientifique, MNHN, SU, EPHE-PSL, UA. Botanique, Paris, France; 10 SciLifeLab-Data Centre, Uppsala, Sweden; 11 FAIRPoints, Gothenburg, Sweden; 12 Institute of Science and Technology Austria, Klosterneuburg, Austria; 13 Computational Neuroscience Imbizo, Cape Town, South Africa; 14 Deep Learning Indaba South Africa, Durban, South Africa; 15 Faculty of Applied Sciences, Delft University of Technology, Delft, the Netherlands; 16 OLS (formerly Open Life Science), Wimblington, United Kingdom; 17 The Alan Turing Institute, London, United Kingdom; Carnegie Mellon University, UNITED STATES

## Abstract

Inclusion at academic events is facing increased scrutiny as the communities these events serve raise their expectations for who can practically attend. Active efforts in recent years to bring more diversity to academic events have brought progress and created momentum. However, we must reflect on these efforts and determine which underrepresented groups are being disadvantaged. Inclusion at academic events is important to ensure diversity of discourse and opinion, to help build networks, and to avoid academic siloing. All of these contribute to the development of a robust and resilient academic field. We have developed these Ten Simple Rules both to amplify the voices that have been speaking out and to celebrate the progress of many Equity, Diversity, and Inclusivity practices that continue to drive the organisation of academic events. The Rules aim to raise awareness as well as provide actionable suggestions and tools to support these initiatives further. This aims to support academic organisations such as the Deep Learning Indaba, Neuromatch Academy, the IBRO-Simons Computational Neuroscience Imbizo, Biodiversity Information Standards (TDWG), Arabs in Neuroscience, FAIRPoints, and OLS (formerly Open Life Science). This article is a call to action for organisers to reevaluate the impact and reach of their inclusive practices.

## Introduction

Discussions of diversity and inclusion are becoming more ingrained in academic event planning with increased scrutiny and expectations from participants, speakers, and organisations. This progress is attributed both to individuals and representative organisations actively listening to those speaking up (for example, the Disability Visibility Project [1] founded by Alice Wong, and Yasmin Benoit’s The Ace Project, facilitated by Stonewall [2]) as well as initiatives intended to amplify these voices (for example, the Diversity Champions Programme by Stonewall [3], DiversityQ [4], and LEAD Network [5]). In the academic event space, there is essential work being done at all levels to prioritise the efforts of Equity, Diversity, and Inclusion (EDI) committees within the planning process. EDI committees work towards, for example, supporting physical access for disabled colleagues, improving global reach through virtual events, broadening language diversity, fundraising to support the participation of underrepresented communities, and developing inclusive communication strategies [6–15]. A previous publication, Ten Simple Rules to host an inclusive conference [7], provides a comprehensive overview of the creation of diverse EDI teams, welcoming environments, improved physical accessibility, and the development of inclusive communication strategies. Many inclusivity practices, such as childcare support and diversity in speakers, that may have been unimaginable a few years ago, are now mainstream event considerations in 2023. However, we need to remain cognisant that there is still much to be done. We need to continue establishing more fine-grained approaches to inclusion at academic events, such as conferences and short courses commonly referred to as “summer schools” [16]. This involves creating platforms for voicing, discussing, and listening to the access needs of those who have historically been excluded from academic events.

We believe, when pushing the boundaries of academic inclusion, there are 5 driving principles: (1) diversity and inclusion in academic events are important for innovation and the development of novel perspectives; (2) successful implementation of inclusivity practices is likely to look different for different communities; (3) equality is a passive process while equity is an active process; (4) systemic change is an iterative process; and (5) all of the aforementioned principles require shared responsibility. We expand on these principles below, before introducing the Ten Simple Rules.

Diversity and inclusion are essential to resilience, quality, and productivity for any setting, including academia [17–21]. Different voices in a welcoming space lead to stronger engagement, more critical discussion, and less centralisation of knowledge, which can only benefit the practices we work to develop. While we advocate for stronger commitments to inclusion at academic events, it is important to acknowledge there will be challenges stemming from power dynamics, cultural differences, expectation misalignment, and/or other reasons not listed here. However, these Rules provide a framework to start a conversation that is aimed at increasing understanding and building support.

The successful implementation of inclusivity practices is likely to differ across communities. It is important to be mindful that successful inclusion of one community may lead to the exclusion of another. For example, there is considerable debate around the inclusivity of virtual events. Recent publications, facilitated by the global shift towards remote attendance during the COVID-19 pandemic, advocate for virtual events as the main solution for improving inclusion [13–15]. While this has overall been a positive shift, we believe that there are undeniable benefits to in-person engagement and that virtual events can be exclusionary to certain communities. Virtual events can be accessible; however, the lack of in-person engagement can contribute to long-term detrimental effects among demographics already experiencing other access-related inequalities [22]. In-person events are important to ensure active engagement and availability of networking opportunities for attendees, which is especially valuable to those less established within the field, such as early-career researchers [23]. While not immune to these negative outcomes, in-person events that prioritise inclusivity can also reduce the formation of academic silos, echo chambers, and the centralisation of knowledge. All of these can lead to top-down development of policies, further excluding underrepresented voices in decision-making.

Equality (ensuring each person is treated the same) is a passive process, while equity (ensuring each person is treated fairly within their own unique context of needs) is an active process (see the Glossary in the S1 Text for the full definitions). We advocate for inclusivity being an active process, which requires consistent effort, transparent decision-making, and for organisers and attendees alike to take responsibility for meaningful representation. For example, considering that allowing anyone to register for an academic event is treating attendees as equal, there would have to be active *exclusionary* efforts to prevent groups from registering. However, having the option to attend in principle and being able to attend in practice are not the same. Making the event more equitable therefore requires an active effort to overcome logistical, economic, and systemic barriers for disadvantaged communities. Organisers should reach out proactively, be allies to overlooked groups, and implement suggested changes. An aspect of allyship (see S1 Text) is creating routes for constructive feedback where input can be relayed without fear of retaliation. Transparent communication of values and efforts to actively champion communities is reassuring for potential attendees. Furthermore, transparency of the inclusion process holds organisers accountable and prevents mere “performative allyship” [24,25]. For example, for attendees, it requires extra effort to inquire about the accommodations and inclusivity values of the event. These inquiries place pressure on the attendees to communicate constructively, navigate privacy concerns, and risk being dismissed as “difficult.”

Systemic change is an iterative process. It requires adaptability, a willingness to repeatedly attempt and then actively learn from failures as steps towards the implementation of ideas for change. In doing so, academic event organisers need to take responsibility for the impact of decisions, especially on those from historically excluded communities. We acknowledge that not every community can be fully represented, nor do organisers have capacity to implement all the possible inclusive changes we propose. However, we advocate for a commitment, by organising teams, to work towards more inclusive events. This commitment will result in a positive step that can help create goodwill and momentum for change.

When pushing for positive change, organisers and attendees should share the responsibility for inclusion, according to their relative power to do so. Historically, organising teams are partially made up of volunteers who carry out a range of time and labour-intensive tasks. Volunteering has advantages for early-career researchers in gaining experience and visibility. Nevertheless, an organising team’s EDI roles are often given to already overworked and EDI-advocating academics [26], often members of underrepresented groups themselves [27]. Given these uneven distributions of labour among volunteer organisers and attendees of the events, it is important that all organisers and attendees learn to step up to support other attendees [28–30]. We acknowledge that there will be finite capacity and resources are limited, but we encourage all stakeholders to push boundaries, mindfully, to own the impact of their decisions, to recognise limits, and to be intentionally bold.

**Fig 1:**
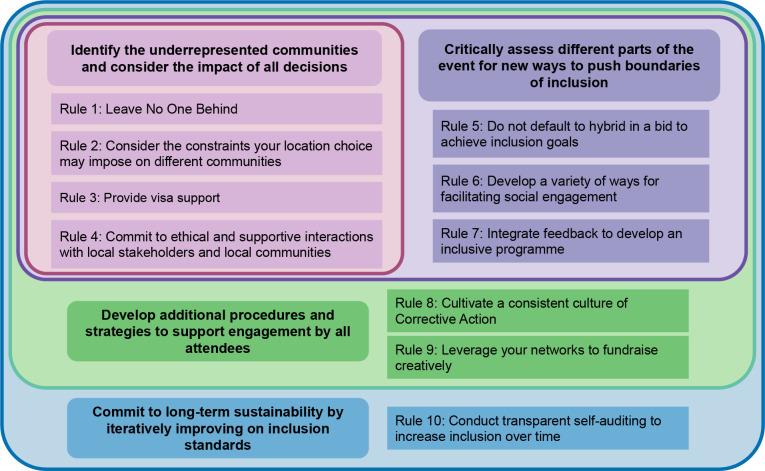
Graphical depiction of the Ten Simple Rules. The Rules should be viewed holistically, and not as a sequential list, nor taken in subsets. We acknowledge there will be different degrees to which each organisation can implement these changes, and we encourage continuous work to compound efforts over time.

Building on the foundation of these 5 principles, we propose the Ten Simple Rules, which we present in 4 embedded layers (see Fig 1). These Rules should be viewed holistically, and not as a sequential list, nor taken in subsets. The first layer is devoted to identifying underrepresented communities and determining if any logistical choices (such as the location) have an impact on these communities and how they can be altered to be more supportive (Rules 1 to 4). Secondly, once efforts are undertaken to ensure as many people as possible are able to join the event, we encourage that time be taken to critically assess whether a virtual or hybrid event achieves the same degree of inclusivity. Further, organisers should carefully consider whether everyone will be able to engage, and whether support is given to local communities surrounding the event (Rules 5 to 7). The third layer presents foundational changes that underpin the Rules: developing inclusive cultures to ensure all are able to engage with the event without fear of exclusion, discrimination, and social misconduct (Rule 8). Additionally, we recommend some creative fundraising procedures to help realise many of the goals outlined in these Ten Simple Rules (Rules 9). Finally, we present frameworks for long-term development of inclusive practices, including tools for implementing change and a self-audit tool for ongoing development of active inclusion in a transparent and iterative manner (Rule 10).

The ideas and practices discussed in these Rules come from those directly impacted by the structural inequalities that currently hinder safe and fully inclusive participation in academic events. These practices are based on lived experiences and our own efforts to support academic organisations such as the Deep Learning Indaba [31], Neuromatch Academy [32,33], the IBRO Simons Computational Neuroscience Imbizo [34], FAIRPoints [35], Biodiversity Information Standards (TDWG) [36], Arabs in Neuroscience [37], and OLS (formerly Open Life Science) [38,39]. While most of these organisations are based in high-income countries, many of us are from low- to middle-income countries and work with geographically distributed communities. The authors hold many identities, and face realities stemming from our various cultural, ethnic, and geographical backgrounds, our gender identities, as well as varying levels of power drawn from financial reality and institutional affiliation.

Building on the collective efforts made over the years, we put forward a call to action with these Ten Simple Rules: to continue pushing the boundaries of academic inclusion. This paper aims to amplify the experiences and voices of underrepresented communities. We offer the words of James Baldwin as a guiding principle for using the Rules [40]: “Not everything that is faced can be changed, but nothing can be changed unless it is faced.”

## Rule 1: Leave No One Behind

“*Cultural change is an iterative*, *evolutionary process*. *How we define in and out groups is malleable*. *It takes courage to create new ways of being and connecting with each othe*r.”*—AZA Allsop*, *MD*, *PhD*

The initial steps towards an inclusive culture are 2-fold: (1) ascertain the underrepresented communities missing from a particular event; and (2) prioritise their inclusion by making meaningful changes in the organisation [41]. “Community” can have many meanings. In this paper, we recognise that communities tend to be distinguished by shared key characteristics, such as geographical location and/or shared identities, norms, and interests. The groupings and needs of these communities are likely to differ across academic fields [42,43]. Understanding communities’ needs is important when we consider potential stressors, such as conflicting power dynamics, culture tensions, and misaligned expectations. Taking the time to understand the communities that should be included means a relationship can be initiated, and trust in the ethos of the organisation can be built. The importance of this trust for supporting self-auditing initiatives is expanded upon in Rule 10. The outreach efforts will simultaneously increase awareness for an academic event and can boost attendee rates (see more references on promoting an event in [44]).

A proposed starting point for developing this understanding is to establish dedicated outreach teams to develop tailored descriptions of a respective community and their needs [45]. This can support the building of relationships with specific stakeholders who can, in turn, act as advisors and communicators. These stakeholders should take on different roles within the community, at different career stages. For example, student-run organisations may be an energetic source for sharing information to younger groups. Affinity groups, for example, Black in AI [46], The Black Women in Computational Biology Network [47], Queer in AI [48], or Arabs in Neuroscience [37], may already exist and can be important allies in the quest for inclusion. These groups can be identified by the outreach team in consultation with community stakeholders.

Well-designed questionnaires (see S2 Text, the pre-event questionnaire), focus groups, and social media platforms can be used to gather information about community needs. Gauging community needs will require multiple methods of engaging different community stakeholders, and different methods of reaching them. It is recommended that multiple platforms beyond email are used: leverage existing phone apps and social media–based groups that are more popular, legal, and accessible in different regions [49]. Over time, a database of information can be collated.

Having worked towards identifying the needs of missing communities, organisers must make a material commitment to support these communities, which includes taking meaningful steps towards changing practices. In working to be more inclusive, organisers are encouraged to keep an open mind, listen to communities, and be ready to work hard to implement changes that are suggested. This support is likely to differ from community to community and, depending on resources, may require time to implement. Some support that may be required is outlined in this paper.

The success of these strategies is evidenced by the ever-growing communities of the Deep Learning Indaba, Neuromatch, and OLS who have adopted these outreach practices. Between 2018 and 2023, the Deep Learning Indaba’s network of country-specific communities (referred to as “IndabaX’s”) has grown from 11 African countries to 37. Neuromatch saw an additional 41 countries across 6 continents represented in their summer school cohorts in its first year. The OLS community was started in 2019 with 49 members from 22 countries, and in 2023, it is represented by over 500 members from 54 countries across 6 continents.

## Rule 2: Consider the constraints your location choice may impose on different communities

*For many members of underrepresented communities, choosing an academic event is not simply a case of identifying a shared interest, and a gap in a calendar. Choosing an event can be an anxiety-inducing process, met with fear and exhaustion over visa application processes, or the inability to leave a country due to existing residential visa constraints. Some attendees may need to go through careful and painstaking deliberation over the safety of a location and any discriminatory laws. Attendees may have to consider access to prayer facilities, stimulation-free zones, and access to culturally familiar restrooms. Attendees with physical disabilities may wonder about their independence at the event venue, and how they might travel around the city without incurring excessive charges for private taxis. In an attempt to address the limitations visas and expenses place on members of the African machine learning community, the Deep Learning Indaba exclusively hosts their annual events in different parts of the African continent. While each location will still impose some restrictions on some attendees, the motivation for each location is to boost support for a local region each year. A location rotation schedule means, over time, more and more of the continent will be supported to attend the event.*

Choosing a location takes 3 dimensions into account: the country, the city, and the venue. Choosing one country over another may benefit some communities over others. For example, the same location may have comparatively friendly visa laws but harsher anti-LGBTQIA+ laws. Taking time to understand these restrictions will allow organisers to work on contingency plans for those who may be excluded. The organisers should be upfront about these restrictions and consider their responsibility to provide reassurance and additional support to those impacted by the choice of location. Some cities may present more opportunities than others. For example, more developed cities with large tourist attractions may be an appealing option for travelling attendees and speakers [6]. Furthermore, they may have the infrastructure to support those with disabilities more easily on public transport, given the increased global pressure for accessible tourism [50,51]. In choosing a destination, there may also be a temptation to avoid certain destinations to make a political statement. Nevertheless, it is important to consider that these decisions can negatively impact local communities who would benefit from attending a location close to home [52]. This topic is covered in great detail in a paper [41] that critically explores the ethics of international conferencing and the decision to host the World Congress of Bioethics in Qatar [53]. Choosing the venue itself is an ongoing area of research, and we refer the reader to existing literature [7,8,54].

In the move towards hybrid events, there have been increased suggestions of local meetups to increase access and limit carbon footprints. However, the value of these meetups will differ dramatically based on location limiting access for some to global networks. This exclusion from a global community can further increase research echo chambers, as proximity can make connections easier.

Any chosen location will impact different communities in some way, and it is important to understand who will benefit and who will be constrained. To make events more inclusive over time, consider a location rotation. Locations should be announced as far in advance as possible so that attendees are given time to plan, and organisers have time to action suggestions presented in this paper, such as providing visa support (see Rule 3) and raising additional funds (see Rule 9).

## Rule 3: Provide visa support

We present the case of an accomplished Nigerian nuclear energy scientist’s difficult and frustrating journey to present his research at a high-profile conference on climate change.

*The event in question was hosted in a country that requires a visa. In Nigeria, it is not simply a matter of making an appointment at the embassy, and hoping the bureaucratic process is completed in time. Applicants face the severe risk of banditry, kidnapping, and death by the Fulani Herdsman and Boko Haram when travelling by road across the country to reach the embassy. Therefore, visa appointments require an expensive flight and overnight accommodation to make it to the appointment on time, on top of expensive visa fees. Despite all this, at least in the case of Nigeria, there is an embassy. Not all parts of Africa have consular representations for all countries. In this particular case study, the scientist only received his visa letter from the academic event a few weeks before it was scheduled to take place, placing immense pressure on him to sort out paperwork in time. This delay, coupled with a delay in funding being approved, resulted in the scientist having to travel to the embassy to ask for special circumstance assistance, days after the conference started. The embassy initially refused to help, and, out of desperation, he paid more money to a third party who was able to get some traction on the process. After more hours lost travelling and 4 flights in as many days, the scientist finally arrived at the conference on the day of his presentation. There was not even time to freshen up at the hotel before he was expected to share his work. While this participant was able to attend in part, despite the lost conference days and the immense stress, there are many cases that are unsuccessful.*

**Fig 2:**
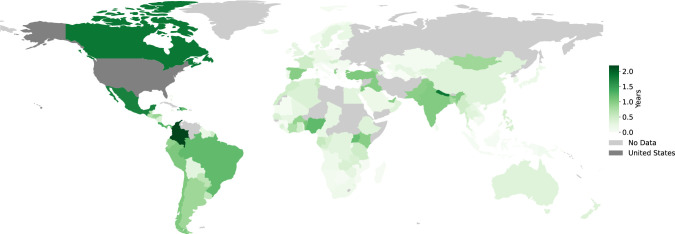
A comparison of processing times to obtain a visitor’s visa to the United States. A map showing the processing times for a United States visitor’s visa in different parts of the world. The scale is in days, with some regions facing a wait time of more than 2 years. From this map, we can identify regions of the world that are significantly more disadvantaged when applying for a visitor visa to the United States. References: [56,57]. Made with Natural Earth. Free vector and raster map data available online at naturalearthdata.com.

**Fig 3:**
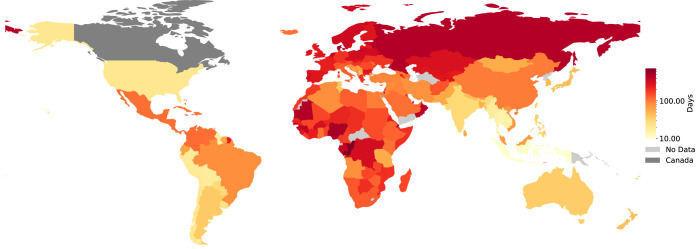
A comparison of processing times to obtain a visitor’s visa to Canada. A map showing the processing times for a Canadian visitor’s visa in different parts of the world. A log scale is used, with the longest wait times affecting the majority of Africa, North Asia, and parts of Central and South America. From this map, we can identify regions of the world that are comparatively more disadvantaged for a visitor visa to Canada. References: [56,58]. Made with Natural Earth.

**Fig 4:**
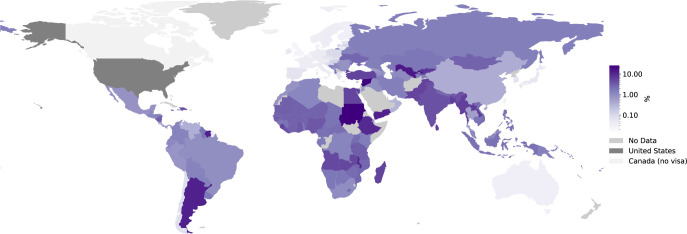
Fees for a visa to the United States as a percentage of annual income. A map showing visa fees for the United States as a percentage of annual income (log scale). From this map, we can identify nationalities of the world that are significantly more disadvantaged when paying visa fees, namely, Africa, South America, the Middle East, and large portions of south and north Asia [56,59]. Made with Natural Earth.

Visa applications are time consuming to prepare (see this eye-opening blog post titled, “In Pursuit of a Visa” by Yanina Bellini Saibene [55]) and can take months to complete (see Fig 2). Visas can be prohibitively expensive, especially when compared to the average annual incomes of different countries (see Figs 3 and 4). Meticulous and accessible visa support is essential as visa rejections can interfere with future visa applications of attendees and thus significantly impact future travel as well as career opportunities.

While we advocate for the identification of locations with friendlier visa access (see Rule 2), in the event that a location is chosen that has strict visa policy, there are different degrees of administrative and financial support that organisers can provide. The first degree of support would be the provision of a visa invitation letter, detailing the academic event and the support the attendee will receive (for example, any travel or accommodation grants). These letters can be automatically generated, and attendees should be able to flag their need for additional support easily on the event website. In addition to administrative support, extra time can be given to attendees to plan. We propose that experiments be conducted in terms of rolling deadlines to prioritise the release of acceptance letters to those needing visas. Announcements about locations should be made at least a year in advance of events, so that timely visa invitation letters can be provided, in the event an attendee is able to purchase their registration ticket.

The second degree of support would be for organisers to send the details of attendees to respective consulates and provide financial reimbursement for expensive visa fees. This process can be facilitated by a pre-event questionnaire that collects information for administrative support and helps attendees understand their responsibilities in ensuring a smooth visa application process (see questionnaire in S2 Text). While direct flights are the best option, they are not always available: Be prepared for the provision of support when it comes to transit visas. Additional fundraising can provide financial support for attendees (see Rule 9). The development of relationships with respective airlines can be helpful as airlines can provide direct communication about assistance for connecting flights and during any unexpected delays.

The third degree of support is the most important in ensuring long-term sustainability of providing visa support. This layer advocates for organisers to invest time in seeking out and developing a partnership with, for example, the Departments of Education, and Interiors, or the equivalent of host countries. Their goals of supporting education and development within their countries align with the goals of academic events. Write a compelling and respectful letter, outlining the following: the description of the event, details about the schedule, the venue, and the attendees that are expected. Advocate for the inclusion of these attendees by including a strong diversity statement and how their presence will contribute to the success of the event and the advancement of the field. Be sure to ask what can be done on the part of the organisers to support this process. Importantly, take the time to build rapport by leveraging a multinational team. Relationships may require time to build, and so the venue rotation, as defined in Rule 2, can help with developing these years in advance.

This rule may not apply to all academic events. For the large proportion of the international scientific community and most contributors of this paper, visa requirements are often the primary concern. While none of these special arrangements can be guaranteed, it is worth investing the time and effort to develop them as part of much needed change to support future academics across the globe [60]. A visa support team, with diverse representation in terms of language and geographical backgrounds, should be established. This team will be tasked with familiarising themselves with the visa support of the respective host country. The work of a visa team can alleviate an immense burden placed on attendees who may be forced to take time off work, organise expensive travel, arrange childcare, and who may deal with language barriers and complicated online processes without prior international travel experience and guaranteed access to the internet and/or electricity. Overall, supporting administrative burdens can make it a much fairer experience for those looking to engage fully in the event despite the existing barriers.

## Rule 4: Commit to ethical and supportive interactions with local stakeholders and local communities

Academic events often depend on other organisations and teams to provide services to support an academic event. Academic events also have the potential to support local vendors and amplify the local culture in a positive manner. However, the impacts on local stakeholders and local communities are rarely considered when organising an academic event. We define local stakeholders as any team, person, or organisation adjacent to planning, running, interacting, or providing a service with academic events. Local communities refers to those living within the local area where the conference is held and may engage with the conference in different ways such as the provision of entertainment or catering. Local staff and workers are a significant source of support for smooth operations at events, ensuring services such as cleaning, setup, providing entertainment and catering for an event. Despite their significant role, these local stakeholders are often the most overlooked and are typically vulnerable to the most harm.

Many workers employed at these events may face job insecurity or a lack of guaranteed pay, being on zero-hour “casual” contracts, or hired through aggregator agencies or platforms. Hiring is typically outsourced, but we propose that academic event organisers leverage their platforms to start questioning unethical hiring practices and demand fair worker compensation and the assurance of safe working conditions. Consultation with local community members and worker unions can aid in the process of uncovering unethical practices and points for improvement. The concerns raised are likely to be different in each location, so due diligence should be employed during every planning stage. It is also important to be aware that due diligence practices should extend not only to the direct contractors but also to their supply chains as well, to the extent possible.

We advocate for the use of local services where possible: Organisers are in a position to empower local creativity and celebrate local pride. For example, organisers using graphic designs and artworks should ensure that local artists are chosen. Local produce, cuisine, and entertainment styles should be prioritised but, at the same time, scrutinised through an intersectional lens to make sure local practices themselves will be inclusive to all attendees and are not likely to cause offence to visiting cultures. The community stakeholders engaged through the proposed practice of Rule 1 can be consulted in this process. All of these decisions to support local solutions can ensure the culture is amplified and work is reimbursed fairly.

The consultations pursued above can aid in the development of organisational standards and criteria to consider when hiring local staff, choosing local vendors, and making decisions around the choice of entertainment and cuisine.

## Rule 5: Do not default to hybrid in a bid to achieve inclusion goals

We present the case of a highly accomplished Humanities academic from Bangladesh.

*In a country as poor as Bangladesh, it is commonplace for scholars to live in a shared house, in a shared bedroom, or in densely packed dormitory-style university accommodation. Academics such as the subject of this case study do not have the privilege of a quiet, private space at home to attend a cognitively demanding academic event hosted virtually. Furthermore, with unscheduled rolling blackouts, as is the reality in many developing countries, stable internet is not a given [61]. This academic is forced to stay late at work, where he is lucky enough to have backup power to engage with events that are in inconvenient time zones for him. Further, the entire experience is isolating, as it becomes more difficult to fully engage with the content and share work due to the lack of privacy, unreliable internet, and fatigue from having to concentrate after hours. For academics in these situations, who come from lower- and middle-income nations where there is not adequate infrastructure to support virtual events, attending in person would be preferable.*

An academic event has 2 types of engagement: engagement with the content and engagement with the network. Choosing a purely virtual format helps increase inclusion by increasing access to content, but it cannot necessarily offer inclusive access to the network. A hybrid event with local meetups can increase access to both types of engagement, and there is a growing body of literature advocating for events to be hosted virtually or as a hybrid event with local meetups [7,12–15]. However, these virtual and hybrid events are still exclusive, in that certain attendees are more likely to be able to attend the main in-person events, and others will potentially be segregated and siloed, depending on their location. Therefore, serious consideration should be given to hosting in-person events, with a commitment to support as many people as possible attending in-person. This is important to avoid the possible segregation of a 2-tier attendee system, with the same people repeatedly attending either in-person or virtually. We would like to challenge the narrative that hybrid events are an “easy solution” to inclusivity and more work needs to be done to make sure some attendees are not eternally relegated to joining virtually. This 2-tier attendee system can contribute to research silos, echo chambers, and increased centralisation of research where established institutions leverage their existing networks to foster development, while opportunities to break into these networks are limited [62].

This Rule is nuanced in that it does not advocate for the total exclusion of a virtual component to academic events but rather calls for the careful consideration of the impact these might impose. It is likely that some attendees simply cannot attend for health, visa, family, or financial reasons, and some might choose not to travel to reduce their overall carbon footprint [63,64]. The pandemic showed us a new age of virtual conferences, allowing content engagement regardless of location or health status, but this is still not the preferred option for attendees in many countries who do not have stable electricity and affordable internet access [33]. We should balance the idea that hybrid events promote some degree of inclusion, primarily for the content, while acknowledging that they are insufficient to provide the same level of access to the academic network as can be facilitated by in-person events [14,15,64].

## Rule 6: Develop a variety of ways for facilitating social engagement

We present the experience of an early-career South Asian academic whose intersectional narrative shaped her dynamic within a conference in a foreign country:

*“Walking into the conference venue made me stop in my tracks. Though initially excited, I now feel anxiety creeping into me. Somehow, I feel hypervisible and invisible at the same time. Not knowing how to converse, I feel trapped in my mind. Questions of whether I am dressed right, if I appear acceptable and how others perceive me cloud my mind. Slowly, I retreat to the corner of the room and take a seat*.

*As the conference goes on, I attend the discussions and talks but slowly notice that the mic is never passed onto me when I raise my hand to ask a question or add a point. A vivid memory of the coffee break pops into my mind when I stood, alone in a corner, sipping my coffee after being brushed off by a senior professor at the table. Later, during the evening social hours, afraid of being deemed lesser, I isolated myself. That is when I noticed a few other attendees, those who were early-career academics and from diverse backgrounds standing around. They seem more approachable and so I muster the courage to begin chatting with them. Slowly, we come together, sharing ideas and discussing our backgrounds and the talks.*

*We keep speaking—it turns out, in our feelings of being invisible, my loneliness was also shared with them.”*

Academic events, particularly those held in-person, can create an atmosphere of engagement and debate. These events are further enriched by networking opportunities. Everyone possesses different levels of confidence and social anxiety, so additional steps should be taken to facilitate these processes. Social anxiety and avoidance of social interaction at an academic event can stem from factors such as uncertainty about the dress code, the time of day of activities, the need to mentally recharge, unstructured social engagements, and differences in customary greetings. Additionally, overindulgence in alcohol can be a leading factor toward inappropriate antisocial behaviour and sexual harassment [65,66].

We propose the development of procedural guidelines that are introduced in pre-event communication. Alongside guidelines, a designated group of volunteers can demonstrate acceptable social greetings and conduct and discuss the dress code and various interpretations thereof that are sensitive to diverse cultural heritages and financial backgrounds. This can be done during plenary addresses and the demonstration thereof can be delivered by the Welcome Wagon [67]. A Welcome Wagon is a welcoming committee formed to support attendees during the planning and attending stage at conferences. Name tags should be provided with the ability to add pronouns and nonverbal signalling, such as stickers and badges with different patterns and lanyards with different fabric. The distinct patterns and fabric can relay a different signal such as greetings an attendee is comfortable with: for example, no contact, handshakes or elbow bumps only, or hugs are welcomed. Colour as a signal should be used sparingly and only after careful consideration to include those that struggle with colour blindness. Badges with pronouns and greeting signals should not be preprinted to allow the attendee to signal as they feel comfortable on the day. Further facilitation of social engagement can be introduced by advocating for diverse approaches to networking, such as breaking cliques with the Pacman rule (where attendees are encouraged to leave an opening in any conversation circles to make more inviting for someone to join) and encouraging diverse engagement with the Snowball rule (where attendees are encouraged to talk to as many people each day as the number of times they’ve attended that particular event) [68,69]. Connecting frequent attendees with new attendees or including a newcomer session in the programme can help increase engagement and lower the barrier to networking, particularly for students and early-career researchers.

Careful consideration should be given to the inclusion of alcohol. The feasibility of total exclusion is debatable, especially depending on context and location. In the event that alcohol is included, we urge caution and consideration of imposing certain restrictions, such as paid bars, limited sponsored tokens, and constrained times for serving alcohol [66,70].

Alternative forms of meetings can be explored, such as “walk and talks” or “*netwalking*.” These walks must take into account different physical capacities and take place in a safe way (for example, by avoiding major intersections and unsafe areas) [71].

Finally, as described in more detail in Rule 7, incorporate changes into the programme to be mindful of religious times and family responsibilities, and make provision of space for alternative forms for recharging, such as quiet, low stimulation zones where social engagement is actively discouraged.

## Rule 7: Integrate feedback to develop an inclusive programme

Conferences and adjacent academic events are known for intense programmes with parallel streams of information packed into a few days. Academic event organisers face a mammoth task in addressing the expectations of a diverse group of attendees. Programmes attempt to aid in delivering keynotes, facilitate discourse, and facilitate networking, all within a relatively short amount of time. In attempting to address all expectations and deliver an engaging programme, there is a high chance of excluding many participants even if they are able to attend the event. The exclusion is most commonly due to an intense programme that becomes a source of major anxiety and pressure for attendees and speakers alike. These feelings of anxiety can be experienced, irrespective of their background and accessibility needs, as all attendees are expected to have high levels of engagement throughout the day to fully utilise the opportunities the conference provides. Missing out on aspects of the academic event programme can lead to increased stress levels due an extensive feeling of “fear of missing out” (FOMO) and guilt over the “wrong” choice [72].

Attendees may struggle to engage fully with the programme for different reasons. These can include, but are not limited to, religious and cultural commitments, care responsibilities, or the need to rest and recharge after a time in an overwhelming sensory and social environment. In order to understand these challenges and constraints, we advocate for seeking feedback from different members of the community in order to better understand the stress-points in the programme. This feedback can be sought during the planning phase and again after the event to inform planning for the next event. We propose that the pre-event questionnaire we introduce in S2 Text be used to assist in this process.

Feedback on the conference programme can inform the development of a programme that is sensitive to the needs of the community. Changes to a programme can include well-timed breaks and fewer parallel sessions with overlapping interests. An inclusive and well-thought through programme will allow time for attendees to engage in their respective responsibilities and take time to recharge without guilt of missing any opportunities.

## Rule 8: Cultivate a consistent culture of corrective action

We advocate for academic environments that are welcoming, friendly, and inclusive so that all participants have the freedom to engage fully without fear for their safety, without having to face discrimination, and/or uncomfortable and distressing encounters. Due to the scale of interpersonal interactions, and the nature of debate and discourse, consistently enforcing a Code of Conduct (CoC) is an integral part of an academic event. Addressing these sensitive matters requires the devotion of significant amounts of time and thought to develop appropriate guidance and practices to support the CoC at an academic event.

We propose the term *Corrective Action* to describe addressing issues or acting to enforce the CoC. We recommend this term, rather than disciplinary action, for example, because corrective actions aim to improve things rather than simply punish and should, ideally, benefit all parties involved. Those reporting a concern or incident should feel heard and those who have behaved in ways that contravene the CoC should be made aware of the consequences of their choices *and* supported to improve. All parties involved should be treated fairly and would ideally agree on the action taken. Approaching matters in this way encourages everyone to take responsibility for the impact of their actions, regardless of initial intention. Corrective action does not aim to encourage “cancel culture” [73,74] but, instead, to encourage a culture of growth and mutual understanding, while empowering people to act as needed to create and maintain safe and respectful spaces.

The consistency of the CoC and corrective action is important [75] and should not be hindered by factors such as the status or relative power balances of those involved. It relies on a team that is dedicated to developing a thoughtful and comprehensive CoC that is put into practice in a mindful manner when issues arise. Training can be especially beneficial to support and empower teams in the promotion of good conduct, as well as how to act when misconduct occurs [76]. Organisers should aim to foster an environment where all parties feel able to advocate for themselves and for others by offering, for example, allyship training and conflict resolution training. It is important to encourage bystanders to either step in or report situations that they feel should be addressed with corrective action [28,77]. Different communication platforms should be leveraged to state the expectations of attendees and organisers to bolster potential bystanders into intervening when they encounter a clearly contravening situation. This can be facilitated through the open dissemination of the CoC, plenary addresses, and the Welcome Wagon (welcoming committee) introduced in Rule 6 to demonstrate and explain expectations and procedures [67].

This development of CoCs should be guided by the process of consultation and iterative improvement to continuously incorporate new lessons as they are learnt. The CoC should be frequently audited to ensure it is, in fact, protecting the communities for which it is designed. To support the development of CoCs, we present the following examples: the Deep Learning Indaba’s code of ethics and conduct (available in 5 languages) [78], the Imbizo’s CoC [79], and the OLS’ CoC [80]. These CoCs rely on clearly defined, visible, and accessible mechanisms for reporting issues, such as anonymous forms and reporting hotlines. The useR! Knowledge base also provides guidelines for establishing CoCs [81].

## Rule 9: Leverage your networks to fundraise creatively

*Drawing inspiration from their community members’ efforts to raise funds through personal “GoFundMe” and similar crowdsourcing campaigns, the Deep Learning Indaba launched a campaign on GoFundMe. This platform was set up to leverage their personal and global networks to actively seek funding to supplement that shared by their generous sponsors. All these efforts are geared towards supporting the Deep Learning Indaba’s mission: to strengthen African AI and support Africa and its people in becoming active shapers of this narrative. Fundraising allows the Indaba to offer full travel and accommodation scholarships to the majority of its students to participate in their annual event, with the explicit goal of extending this support to all students in future.*

**Fig 5:**
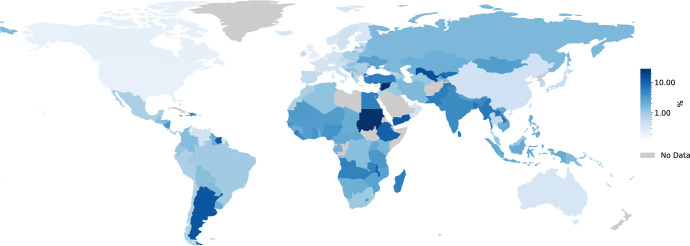
Conference fees as a percentage of annual income globally. This shows a 200 USD conference fee as a percentage of annual income, in log scale. This map also shows regions disadvantaged by the pricing, with significant overlap to the regions already disadvantaged by the visa fees. References: [56,59]. Made with Natural Earth.

Fundraising is a standard part of organising most academic events [6,82–84]. Success relies on the leveraging of personal connections, a strong fundraising strategy, and alignment between stakeholders and the mission of the organisation. This alignment of the mission can help draw in donations and sponsorship directly or build a network of people helping spread the message. To enable participation of diverse academic actors through equitable access to resources, organisers should plan with the mindset that financial support is essential to increase the inclusivity of events. For many, even the lowest of conference fees are prohibitively expensive (see Fig 5). We would like to propose for organisers to go beyond standard measures and start thinking creatively about less traditional means of fundraising such as organisation-driven crowdfunding. Importantly, this also comes with the responsibility to maintain transparency regarding how crowdsourced funding is handled.

Organisations typically have influential social media platforms that can be used not only to amplify the voices of those who are almost certainly fundraising themselves but also—as inspired by the Deep Learning Indaba organisation—to run crowdfunding campaigns to support attendees. This allows the connections of friends, family, and community members to be leveraged, along with entities that may not have the legal or financial capacity to donate funds through official sponsorship channels. This route, while encouraged, will require due diligence from the organising team to investigate whether the “GoFundMe” platform or similar is supported in the respective region [85]. As mentioned above, the success of these campaigns relies on developing a personal connection between donors and the organisation’s mission [84]. Accounts of individuals and organisations can create a compelling narrative to support this alignment by highlighting the impact of either attending or not attending in-person. Therefore, it is important to collect stories of both successful attendees and those who could not attend for financial reasons. This can be facilitated by pre- and post-attendance questionnaires (see S2 Text pre-event questionnaire) and used to create powerful and persuasive messaging.

## Rule 10: Conduct transparent self-auditing to increase inclusion over time

We advocate for clear and consistent self-auditing as a means to quantify, evaluate, and demonstrate actions and changes made in the planning of an academic event to support increased inclusion. This self-auditing can be facilitated in at least 2 ways: (1) through engaging with attendees (participants and speakers alike) during the event; and (2) through the use of tools such as the Academic Community Equity (ACE) Index, which we introduce in S3 Text.

Attendees can help in understanding the atmosphere at the event and identify any consistently excluded groups. Before engaging with attendees to seek feedback, organisers should explain that attendees may be approached about their experiences, that constructive criticism is welcomed, and that all information will be held in confidence. If the correct tone is set, attendees should feel empowered to approach organisers with their own observations during the event. This crowdsourcing can help an overburdened organising team identify their weaknesses or help reveal shortcomings they may not have noticed having been so closely involved in the organisational process. Other platforms exist as well, such as BiasWatchNeuro, which is dedicated to tracking speaker diversity (particularly focusing on gender) at different academic events [86]. This platform is supported by a large open community of contributors who submit information to be collated on the BiasWatchNeuro website.

Secondly, to facilitate both the audit and communication, we have developed an extensive and actionable auditing tool (see S3 Text Academic Community Equity (ACE) Index) to help assess the inclusivity of the event both as an attendee and as an organiser. The ACE Index can be implemented in multiple ways. We propose assigning members of the organising team to critically assess the academic event. Promoting the use of the ACE Index at an organisation level on social media and official communication with attendees can raise awareness of its existence. This will contribute to accountability and transparency and support the explicit encouragement of attendees to submit their feedback. A collation of the outcomes of the ACE Index can help inform the next stage of planning of an academic event and contribute to tangible, iterative change in an event’s commitment to inclusivity. Change cannot happen overnight, but time must be spent acknowledging the shortcomings and working towards improvements. Transparent self-auditing is essential in this process.

## Conclusions

Individuals involved in research and the field of research as a whole can benefit from rigorous debate, social networking, challenges, praise, and critique from the myriad of voices found in a diverse group of attendees of academic events. Well-organised, welcoming, and inclusive in-person engagement that is equity centred can facilitate the benefits above. In pushing the boundaries of academic inclusion, it is important to remember that some participants will have fewer barriers to engaging fully in these events, while others will experience significant challenges including lack of funding, visa requirements, disability, health and care responsibility, or active exclusion based on their identity or background. For many underrepresented communities, the latter is too often the reality.

When considering how inclusion can be improved, it is important to work with underrepresented communities and commit to providing support through identifying their needs (Rule 1). Carefully consider the constraints the chosen location will impose, and how it might affect those requiring visas, accessibility arrangements. Special attention should be paid to the laws that discriminate against the underrepresented communities (Rule 2). Once the location is chosen, with mindful consideration of the impacts on different communities, set up a team to help with visa support by understanding the processes involved and engaging with consulates and ministries (Rule 3). Further, commit to ethical engagement and support of the local communities and third parties that will support the event (Rule 4).

Consider whether a virtual or hybrid event will actually help to achieve goals of inclusion (Rule 5) and develop strategies to improve in-person engagement in the event (Rule 6). When developing the schedule, be sure to engage with all attendees to gather feedback on the programme (Rule 7).

The success of all the Rules relies on engaging meaningfully with all stakeholders. This includes the development and consistent implementation of CoC practices, irrespective of the scale and power dynamics involved (Rule 8). Many of the changes suggested here, from hiring administrative staff to facilitating visa support, require additional funds to enable economic equity. Fundraising can be done in creative ways, such as leveraging an organisation’s extensive networks to crowdfund (Rule 9).

Ensuring systematic and sustainable change requires committing to long-term sustainability by iteratively improving on inclusion standards. Build a consistent ethos with transparent self-auditing of the organisation’s efforts to increase inclusion over time (Rule 10).

To facilitate the adoption of these rules within organisational practice, we introduce several tools to support organisers: a pre-event questionnaire (S2 Text), the Academic Community Equity (ACE) Index (S3 Text), an Action Plan (S4 Text), and a Glossary of Useful Terms (S1 Text). The pre-event questionnaire is intended to support the gathering of information about the needs of attendees, which should be incorporated as feedback for the planning of an academic event. We introduce the ACE Index as the means for auditing academic events. This Index can be used by organisers themselves to self-audit their own events as well as by attendees to gauge the amount of support an academic event will offer them. Further, we introduce an Action Plan, which provides a set of discrete steps to be followed in order to implement the proposed changes detailed in these Rules. Finally, to ensure the language used in this paper and other EDI-related publications are accessible, we present a Glossary of Useful Terms.

We realise issues of inclusion are nuanced, that not every event can cater to everyone, and that some rules simply cannot be fully implemented in all environments. However, academic event organisers have the power to push the boundaries of inclusion. They hold the responsibility to improve their processes and adapt procedures to push their perceived limits of inclusion. Change may not happen overnight; however, a commitment to enact radical inclusion in all academic events can motivate the iterative improvement, coupled with rigorous and transparent self-auditing.

We call upon organisers and attendees of academic events to consider these insights, Rules, tools, principles, and suggestions. This article reflects evolving attitudes toward inclusiveness at academic events and raises the standard which organisers (and attendees) should meet. Ultimately, we are all striving to advance our respective fields. We, the authors, are staunch believers that working together gets us further; we have seen it in the events with which we are involved and in the writing of this paper. By striving together, we look forward to seeing new people and ideas flourish in both established and emerging communities.

## Supporting information

S1 Text Glossary of useful terms. (PDF)

S2 Text Pre-event questionnaire. (PDF)

S3 Text Academic Community Equity (ACE) Index. (PDF)

S4 Text Action Plan. (PDF)

S5 Text Lay summary. (PDF)

S6 Text Alternative text for the figures. (PDF)
