# Supplementary material for: Ten simple rules for pushing boundaries of inclusion at academic events
Source: PLoS Comput Biol. 2024 Mar 1;20(3):e1011797. doi: 10.1371/journal.pcbi.1011797 (PMC10906823; doi:10.1371/journal.pcbi.1011797)
Supplement: S1 Text — (PDF) [file pcbi.1011797.s001.pdf]

Glossary Of Useful Terms

Below are terms that have been used in the main text and/or are frequently used in the literature relevant to this work.

- **Accessibility:** The design and implementation of academic events to accommodate the needs of all attendees, including those with disabilities, to ensure equal access to information and participation.
- **ACE:** Academic Community Equality Index - a benchmark created here to assess inclusion at academic events with the intention of acknowledging success and improving other areas.
- **Allyship:** A commitment by individuals to support and advocate for marginalised or underrepresented groups, working to challenge systemic biases and promote inclusivity.
- **CAKE:** Principles that academic conferences can aim to achieve in the spirit of FAIR data principles [1].
    - Connectivity: Highlights the importance of connecting with people and ideas at conferences. This involves creating opportunities for attendees to network, share insights, and establish meaningful connections. Offer interactive workshops, discussions, and networking opportunities to ensure that attendees are actively engaged.
      "Is everyone seen"
    - Adaptability: Recognise the need for flexibility and adaptability in conference planning and execution. Pertains to making conferences and interactions accessible and relevant to a diverse range of attendees. This includes providing arrangements, promoting inclusivity, and ensuring that barriers to participation are minimised. Create an environment where attendees and organisers can adjust their approaches and ideas based on evolving circumstances.
      "Is everyone themselves"
    - Kinship: Emphasises the cultivation of a sense of connection, community, and shared purpose among conference participants. This involves fostering a feeling of belonging and mutual support, encouraging attendees to forge meaningful relationships and collaborative endeavours that extend beyond the conference setting. Similar to Connectivity, Kinship highlights the interpersonal bonds and common goals that are nurtured through conference interactions.
      "Does everyone feel like they belong"
    - Empowerment: Empower attendees to take ownership of their learning and experiences. Provide resources, tools, and support that enable attendees to make the most of their time at the conference and apply what they've learned. This could involve follow-up activities, collaborative projects, or ongoing dialogues to ensure that the value of the conference extends beyond its duration.
      "Can everyone (and the community) grow?"
- **Cultural Competence:** The ability to effectively interact with individuals from different cultural backgrounds, demonstrating awareness, understanding, and respect for diverse perspectives.

- **Cultural Sensitivity**: Being aware of and responsive to the cultural norms, practices, and sensitivities of different groups, creating a respectful and inclusive environment.
- **Diversity**: The presence of a wide range of individuals, encompassing various identities, backgrounds, cultures, and perspectives, within a particular setting or environment. Embracing diversity fosters an inclusive atmosphere that values and respects the unique characteristics and contributions of each person, enriching collective experiences and understanding.
- **EDI:** Equity, Diversity, and Inclusion. Also abbreviated as DEI.
- **Empowerment:** Enabling individuals to assert themselves, make informed choices, and actively contribute to academic discussions, fostering a sense of agency and ownership.
- **Equality:** Ensuring each person is treated the *same*, especially in terms of rights, opportunities, and outcomes, regardless of their backgrounds or identities. This is easily achieved by merely being passive about who attends.
- **Equity:** Ensuring each person is treated fairly within their own *unique context* of needs and circumstances. This involves acknowledging and actively addressing historical injustices and supporting underrepresented groups and individuals to help them achieve the same outcomes.
- **Implicit Bias:** Unconscious attitudes or stereotypes that affect our understanding, actions, and decisions, potentially leading to unintended discrimination or exclusion.
- **Inclusion**: The practice of actively involving and valuing diverse individuals, perspectives, and backgrounds in academic events, fostering a sense of belonging and equitable participation.
- **Interdisciplinary:** Incorporating multiple academic disciplines or fields of study within events, encouraging cross-pollination of ideas and perspectives.
- **Intersectionality:** The recognition and consideration of how multiple dimensions of identity (such as gender, race, class, and more) intersect and influence experiences, contributing to a more nuanced understanding of inclusivity.
- **Linguistic Diversity:** Acknowledging and respecting the diverse languages, dialects, and communication styles that participants may bring to academic events, enhancing cross-cultural understanding.
- **Microaggressions:** Subtle, often unintentional, acts or comments that communicate bias or insensitivity toward individuals based on their identities, contributing to a less inclusive environment.
- **Representation:** The presence and visibility of individuals from diverse backgrounds in various roles and positions within academic events, promoting a broader range of voices and experiences.
- **Safe Space:** A supportive and welcoming environment within academic events where participants can openly express their ideas, opinions, and experiences without fear of judgement or discrimination.

Reference

1. Wilkinson MD, Dumontier M, Aalbersberg IjJ, Appleton G, Axton M, Baak A, et al. The FAIR Guiding Principles for scientific data management and stewardship. Sci Data. 2016 Mar 15;3(1):160018.
