# Supplementary material for: Ten simple rules for pushing boundaries of inclusion at academic events
Source: PLoS Comput Biol. 2024 Mar 1;20(3):e1011797. doi: 10.1371/journal.pcbi.1011797 (PMC10906823; doi:10.1371/journal.pcbi.1011797)
Supplement: S2 Text — (PDF) [file pcbi.1011797.s002.pdf]

Pre-event Questionnaire

This is an example of a pre-event questionnaire that can be shared with participants. These questions can be adapted for the purpose of different events. The Pre-event questionnaire should be shared with attendees who are likely to attend, and those that indicate they cannot attend anymore. Please adapt the questionnaire to suit your needs and clearly indicate the options that are possible, and which are to support future planning efforts. Phrases such as the following can be used: “If the following were to be offered, would you be more likely to attend?”

This questionnaire is not intended to be used as a survey to understand the demographics of attendees and experiences during the conference, but rather solely focuses on ensuring inclusivity in event planning.

*[** Comments are included in grey and between square brackets]*

Personal Details

- Name:
- Title / honorific (optional):
- Affiliation:
- Email/ (or other method of communication if such is preferred):
- Nationality:
- Country of Current residence:

Attendance

- Are you planning to attend this event?

- If not, please check the boxes that correspond to the reasons you are unable to attend.
  - Financial reasons (*for example, I do not have the budget to book the flights and accommodation)*
  - Family or dependant responsibility (*for example, I am a primary caregiver for young children)*
  - Visa (*for example, I am unable to secure a visa due to the associated costs being too high, or perhaps there is a lack of consular representation)*
  - Unsuitable location *(for example I do not feel safe at the location due to my identity)*
  - Health concerns *(for example, I do not feel comfortable attending the event at a mask-less venue in high disease-transmission zone)*
  - The content of the conference *(for example the invited keynote speaker has an unacceptable and vocal position on an issue for my community)*
  - Other (please specify)

- If you would like to share additional details on the reasons provided above, we would appreciate the feedback so we can work towards providing additional support in the future.

Dietary Requirements

- Do you have any dietary requirements? These could potentially relate to allergies, food intolerances, religious requirements, or personal reasons (please specify as many as needed).
  - Please note it is unlikely we will be able to cater for individual dietary preferences, but this information will help us estimate need and label food appropriately and clearly to help you make informed decisions.

- Lactose intolerant
- No eggs
- No fish
- No shellfish
- Tree nut allergy
- Peanut allergy
- No wheat
- No soybeans
- No sesame
- No onions
- Vitamin B12 allergy
- Vitamin D allergy
- No grapefruit products
- No fruits
- Coconut allergy
- Avocado allergy
- Vegetarian
- Vegetarian (excluding eggs)
- Vegan
- Halaal
- Kosher
- No alcohol
- No pork
- Other (please specify)

- Would you require any dietary arrangements for the dependants travelling with you (*such as formula milk or soft food*)?

Accessibility Needs

- Are there any accessibility adjustments that can be made to support you at this event?
- What accommodations have worked well for you in the past?
- Is there anything else we can do to make your experience more accessible?

Financial Needs

- Are you applying for financial assistance to attend this event?

- If yes, please provide a brief description of your financial need.

Visa Needs

- Would you require a visa to attend the full length of the conference?

- If yes, please provide the visa application deadline and any other relevant information.

- Please select all that apply. If any of these are no, please note you may need to apply for renewal:
  [These questions are designed to help attendees, especially those that will be travelling for the first time, understand what is required of them to ensure a smooth visa application process]
  - Do you have a passport as per your country's government standard? Please use your citizenship country rather than residence one, if these are not the same
  - Will your passport expire more than six months post the event?
  - Does the passport have at least two blank pages?
  - Does your passport have correct details, dates and is it free from serious damage (passports may be considered invalid if drawn in, torn, have typos or inconsistencies with other documents)?

The Schedule

In this section please add the available sessions in the proposed timetable to understand the following:

- What is the predicted attendance at each of the sessions (*for example, please highlight the sessions that you are planning to attend*)?
- If multiple streams are present, any potential conflict or will you end up with a packed schedule (*for example, looking at the current timetable, please highlight the sessions that are currently in conflict for you*)?
- Are there any other scheduling or preference restrictions that are required (*for example, in the following empty calendar, please block off the times that you would be unavailable to attend the conference events including but not limited to family commitments, religious commitments, and/or medical commitments*)?

Have you taken time to read through and familiarise yourself with the code of conduct?

- Yes (compulsory)
- Note (optional): please add any considerations that the organising team should be aware of when it comes to appropriate conduct during the event (*for example, I am an attendee with Tourette syndrome and some of my tics rarely include obscene movements or gestures (copropraxia))*

Are you willing to be contacted at a later date to share a testimonial?

This is to help us reflect on our practices, and help collect stories to motivate our actions as well our fundraising efforts. We would like to learn what we are doing well, and what that means to you, as well as learn our shortcomings and the negative impacts of those. Testimonials can be used on our website, for external reporting and in external presentations. This is different from feedback, which is given fully anonymously and will only be used to improve the future experience and for internal statistics.

- Yes, anonymously
- Yes
- Please ask again at a later date
- No

Any other information
Please use this space to provide any additional information that you would like us to know. Thank you for your time and consideration.
