# Supplementary material for: Ten simple rules for pushing boundaries of inclusion at academic events
Source: PLoS Comput Biol. 2024 Mar 1;20(3):e1011797. doi: 10.1371/journal.pcbi.1011797 (PMC10906823; doi:10.1371/journal.pcbi.1011797)
Supplement: S3 Text — (PDF) [file pcbi.1011797.s003.pdf]

Academic Community Equity Index (ACE)

This benchmark is proposed as a starting point for the auditing of academic events. This tool can be used by organisers as a way of self-auditing and ensuring that decision-making is more transparent. This tool can also be used by participants as a way to score academic events to determine if they feel supported enough to attend. It is a simple binary scoring system, and the higher the score, the better the academic scores in terms of its commitment to inclusivity.

This benchmark is a work of ongoing development and you can submit suggestions in the GitHub repository: https://github.com/smhall97/academic-community-equity-tools

**Identifying and providing support for underrepresented communities**	
	Have you provided this support?
Have you engaged with existing communities or encouraged the formation of a community if none exist?	Yes / No
Have you ensured the community members are engaged with and given the opportunity to share insight and feedback during every stage of the planning process?	Yes / No
Have you identified and worked with respective affinity groups?	Yes / No
Have you identified and worked with student community groups?	Yes / No
Have you investigated alternative methods of reaching groups (Whatsapp, Facebook, Twitter/X)?	Yes / No
Score	/5
**Location**	
	Have you provided this support?
Country	
Have you investigated the visa restrictions	Yes / No

related to this country?	
Have you investigated the legal restrictions related to this country and the LGBTQIA+ community?	Yes / No
Have you investigated conflict, economic or political climate related to this country?	Yes / No
City	
Have you investigated public transport accessibility?	Yes / No
Have you investigated a range of local hotel costs?	Yes / No
Have you investigated the accessibility of travel from other / cheaper neighbourhoods?	Yes / No
Venue	
Have you investigated the physical accessibility of the venue?	Yes / No
Have you investigated the sensory accessibility of the venue?	Yes / No
Is there a clear sign-posted and accessible space for religious practices?	Yes / No
Are you able to offer or fund child care support at or near the venue?	Yes / No
Are there accessible quiet spaces for restful relaxation or sensory recovery?	Yes / No
Score	/11
**Provide visa support**	
	Have you provided this support?
Have you established a visa administrative team?	Yes / No
Have you been providing timeous visa letters to the attendees?	Yes / No
Have you sent information about visa	Yes / No

applicants directly to embassies?	
If visa applicants information was collected, has it been kept and processed with strict compliance to data privacy laws?	Yes / No
Have you offered visa reimbursement schemes?	Yes / No
Have you attempted to reach out to partner with Ministries of Education and Interiors, or the equivalent in host countries?	Yes / No
Have you managed to partner with Ministries of Education and Interiors, or the equivalent in host countries?	Yes / No
Score	/7
**Planning the schedule**	
	Have you provided this support?
Have you enquired about the scheduled commitments of attendees (such as childcare, religious commitments)?	Yes / No
Have you enquired about the suitability of schedule for attendees and about possible conflicts?	Yes / No
Have you enquired about attendance for the planned sessions to ensure appropriate space allocation?	Yes / No
Have you provided support sessions for first-time attendees?	Yes / No
Have you provided welfare sessions for attendees?	Yes / No
Have you planned allyship training sessions throughout the day and ensured an active engagement from attendees?	Yes / No
Score	/6
**Facilitating Social Engagement**	
	Have you provided this support?

Have you provided and made attendees aware of the name tags with non-verbal cues and optional pronouns?	Yes / No
Have you communicated the dress code clearly?	Yes / No
Have you made attendees aware of and implemented the Pacman rule?	Yes / No
Have you made attendees aware of and encouraged breaking of cliques with the Snowball rule?	Yes / No
Have you ensured there are alternative social engagements?	Yes / No
Have you ensured there are communication and networking options for attendees after the conference?	Yes / No
Score	/6
**Fundraising**	
	Have you provided this support?
Have you shared attendees' fundraising efforts with the community and actively amplified these?	Yes / No
Have you established a crowdfunding campaign?	Yes / No
Score	/2
**You have reached the end of the benchmark**	
