# Supplementary material for: Ten simple rules for pushing boundaries of inclusion at academic events
Source: PLoS Comput Biol. 2024 Mar 1;20(3):e1011797. doi: 10.1371/journal.pcbi.1011797 (PMC10906823; doi:10.1371/journal.pcbi.1011797)
Supplement: S4 Text — (PDF) [file pcbi.1011797.s004.pdf]

Action Plan

This tool is aimed at helping people engage with the content of this Ten Simple Rules paper in a way that does not involve massive bodies of text. The action plan details steps that can be taken to put the Rules into action. Please adapt the content and/or design/format of this sheet to suit your needs.

Each Rule is accompanied by a question, an action, a rationale and the chance for reflection:

- The **question** is set up to help organisers identify a problem, or point for action within the organisation
- The **action** is a set of concrete steps to tackle these points to get started on an inclusive event planning journey.
- Providing a brief **rationale** for the action can help with decision-making and may be useful in justifying e.g. costs to funders. We have provided the rationale for the actions listed, but we encourage adapting these as needed.
- The empty space next to **"reflections"** is designed to be used by you in this process to help you reflect and make choices about what matters in planning your event.

**Rule 1: Leave No One Behind**	
**Question**	Who is consistently absent from either organisation teams and/or attendees at events you have been to? What barriers can you remove or reduce? How will you do this?
**Action**	Ask organisers of previous events to share their learning; reach out to your organisation(s) and relevant local community groups to start inclusive conversations. Make sure that expectations, rewards and limits are clearly set out.
**Rationale**	Having stakeholders, especially those who have not previously been involved, or have adverse experiences can highlight concerns and existing barriers for further actions.
**Reflection**	

**Rule 2: Consider the constraints your location choice may impose on different communities**	
**Question**	Think back to the conference and event locations you have attended and/or helped to run an event at. What informed your/organiser choices? What worked well and what might you change?

**Action**	Research the accessibility and any other restrictions for a previous location - for example, a conference. Identify one thing (or more) to prioritise in your location choices.
**Rationale**	Identifying gaps and good practice from previous events can help you begin to shape your inclusive planning.
**Reflection**	

**Rule 3: Provide visa support**	
**Question**	Are you aware of documents and procedures to support visa applications for attendees?
**Action**	Extend your conference timelines, in particular between notification of abstract acceptance and payment of any fees. Find out how else you can/are allowed to support applications and where to share information.
**Rationale**	Earlier planning and content review allows additional time for the attendees to organise necessary visa or travel arrangements and for organisers to plan with contingency.
**Reflection**	

**Rule 4: Commit to more ethical and supportive interactions with local third parties and communities**	
**Question**	What do you know about the services and suppliers (such as catering, IT teams) at your organisation? What is relevant to your inclusion-centred planning?
**Action**	Think about current suppliers or in-house procedures before your main planning stage – can you source more ethical and sustainable suppliers or make requests from in-house parties (based on, for example, reusable resources, waste management, staff/employment conditions).

**Rationale**	To ensure inclusivity, it is essential to identify relevant issues early and allow time to ask questions, make changes or request adaptations in pursuit of ethical and sustainable working.
**Reflection**	

**Rule 5: Do not default to hybrid in a bid to achieve inclusion goals**	
**Question**	Reflect on your experience of online events: have there been disruptions (such as unstable internet, family commitments, sound or technical issues) and how were they managed?
**Action**	Consider who is joining and/or contributing online and understand what it would take to facilitate their in-person attendance (for example, health-related requirements such as masks, travel support and/or childcare). Assess online platforms for accessibility.
**Rationale**	Heavily relying on online or hybrid may not be the best solution for inclusivity. If it is chosen, we must still think carefully about making it inclusive.
**Reflection**	

**Rule 6: Develop a variety of ways for facilitating social interaction**	
**Question**	Reflect on social engagements at events you and/or others have attended. What worked and why, and how can you change or improve things that did not work?
**Action**	For example, include writable name tags with an option to add pronouns and non-verbal signalling; provide quiet spaces.
**Rationale**	This demonstrates inclusiveness and flexible approaches to interactions, which then creates a basis to expand this approach to all aspects of your event and planning.
**Reflection**	

Rule 7: Integrate feedback to develop an inclusive programme	
**Question**	Reflect on the schedules of in-person events: Were there sufficient breaks? Were sessions a good length? How accessible was the schedule itself?
**Action**	For example, plan additional breaks, allow time to transfer between spaces, mix up formats e.g. talks and workshops. Consider making some resources available after the event.
**Rationale**	This ensures attendees with different needs have the opportunity to recharge and engage fully and to access materials post-event as needed.
**Reflection**	

Rule 8: Cultivate a consistent culture of Corrective Action	
**Question**	Does your institution or event have a Code of Conduct? If yes, is it obvious and accessible for attendees to be able to read and enact?
**Action**	Put together a Code of Conduct if there is not one, or review the existing one with your event/audience in mind. Consider providing training resources on, for example, allyship to the organising and event managing team.
**Rationale**	Majority of concerns can be addressed early on by ensuring everyone knows what is expected, how to report or respond to misconduct at the event and how to best support those affected.
**Reflection**	

Rule 9: Leverage your networks to fundraise creatively	
**Question**	Reflect on the funding provided for the event. How was it secured and what inclusion plans can it support? Is there a way to ensure you can fund further inclusion efforts in the future?
**Action**	During event feedback, try to collect stories from both those who attended and, where possible, those who could not make it (for financial or other need-based reasons).

**Rationale**	These stories can inform funding applications and form a compelling narrative to support development of more personal connections with potential donors.
**Reflection**	

**Rule 10: Conduct transparent self-auditing to increase inclusion over time**	
**Question**	How will you assess inclusion efforts during planning and, if possible, at the event? How will you implement actions for any immediate needs? Who will be responsible?
**Action**	Ensure attendees are aware of ways to communicate with organisers before and during the event (assuring confidentiality where needed). Consistently meet criticism and suggestions with acknowledgement and, where appropriate, actionable steps. Draft a response plan.
**Rationale**	Building a reputation with consistent, transparent and reflective work begins with providing the ability to criticise and suggest changes to the event and responsibly enacting the next steps.
**Reflection**	
