# Supplementary material for: Ten simple rules for pushing boundaries of inclusion at academic events
Source: PLoS Comput Biol. 2024 Mar 1;20(3):e1011797. doi: 10.1371/journal.pcbi.1011797 (PMC10906823; doi:10.1371/journal.pcbi.1011797)
Supplement: S5 Text — (PDF) [file pcbi.1011797.s005.pdf]

Lay Summary

Academic research and related events can be inaccessible in a range of ways and historically have not been designed to accommodate a diverse audience with varying needs. Academic events such as conferences are important for career development and sharing research, but their inaccessibility negatively impacts those who are excluded from attending/being able to fully engage. Thus, it is essential for stakeholders in such events to be aware of and act to address these inequalities from the early stages of design and planning through to delivery and beyond. In this article we recognise existing efforts to improve fairness and diversity, including access modifications to the physical environment, using virtual event options and communicating in inclusive ways. However, more work can be done to make events accessible and welcoming to those from historically underrepresented communities. Here, we offer guidance for working towards 'inclusive by design' academic events, presented as 'Ten Simple Rules', or topics for consideration, during planning, delivery and beyond.

1. Be aware of, and work with the communities that you do not often see at your events.
2. Carefully consider the event location and what access restrictions the location or venue may impose, and on whom.
3. Research and provide support as appropriate for participants to enter the country without excessive costs and stress.
4. Make ethical and sustainable decisions regarding people involved in delivering events, from suppliers to staff.
5. Use virtual or hybrid event options as an additional not an alternative means to greater inclusivity at in-person events and address digital exclusion challenges.
6. Ensure social events are varied and include a range of activity options for people with different needs and tolerances.
7. Ask potential attendees for their contributions at planning stages to make schedules accessible to all.
8. Communicate fair, clear, and actionable expectations with regards to behaviour which are consistently applied and easy to access for attendees.
9. Plan ahead to raise funds for improving inclusion at future events and support other's efforts too.
10. Consolidate learning through reflection and sharing knowledge and be open to constructive criticism from all stakeholders. Finally, ensure that relevant/appropriate changes are enacted where possible and clearly communicated.
