# Supplementary material for: Ten simple rules for pushing boundaries of inclusion at academic events
Source: PLoS Comput Biol. 2024 Mar 1;20(3):e1011797. doi: 10.1371/journal.pcbi.1011797 (PMC10906823; doi:10.1371/journal.pcbi.1011797)
Supplement: S6 Text — (PDF) [file pcbi.1011797.s006.pdf]

S6: Alternative text for the figures

Fig 1. A depiction of the Rules divided into four embedded sections, grouping the Rules into related categories. There is no direction or flow to the Rules as they are meant to be viewed holistically and not as a linear list. Rules one to four are categorised by the heading “Identify the underrepresented communities and consider the impact of all decisions”. The second group is placed to the right of the first group. This group is categorised by the heading “Critically assess different parts of the event for new ways to push boundaries of Inclusion” This includes Rules five to seven. Both of these Rules categories are embedded in a new layer which contains Rules eight and nine under the category heading, “develop additional procedures and strategies to support engagement by all attendees”. Finally, both of these layers are embedded in the all-encompassing layer which contains Rule ten under the heading “Commit to long term sustainability by iteratively improving on inclusion standards”.

Fig 2. A world map showing the processing times for a United States visitor’s visa. This shows the wait time that someone will face in another country to obtain a visa to visit the US. The darker the colour the longer the waiting time. In this plot, we show that the highest wait times of more than two years are faced in regions including Canada and Latin and South America as well as parts of Africa and East Southeast Asia. The United States is depicted in dark grey as visas are not required for this region. Regions without data are indicated in light grey and include parts of North Africa, the Middle East and North Asia.

Fig 3. A world map showing the visa processing times for a Canadian visitor’s visa in different parts of the world. A log scale is used and this shows that the longest rating times affect regions of Africa, North Asia, as well as Central and South America.

Fig 4. A world map showing fees for the United States as a percentage of annual income in log scale. The data highlights Africa as the most impacted region, with parts of the Middle East, South and North Asia and South America also affected by visa fees being disproportionately significant relative to the annual income.

Fig 5. A world map of a 200 USD conference fee as a percentage of national annual income. The majority of countries with the highest percentage, between 20 and 40%, are located in the African and Southeast Asian regions. Other parts of the world do have higher percentages with regions in North America, Europe and East Asia showing the lowest percentages of annual income, between 0% and 10%.
